# Primary malignant mixed müllerian tumor of the peritoneum a case report with review of the literature

**DOI:** 10.1186/1477-7819-9-17

**Published:** 2011-02-04

**Authors:** Fisnik Kurshumliu, Helle Rung-Hansen, Vibeke Ravn Skovlund, Lumturije Gashi-Luci, Thomas Horn

**Affiliations:** 1 Institute of Anatomic Pathology, Medical School, University Clinical Center, Prishtina, Kosovo; 2 Department of Gynecology and Obstetrics, Herlev University Hospital, Copenhagen, Denmark; 3 Institute of Anatomic Pathology, Herlev University Hospital, Copenhagen, Denmark

## Abstract

Malignant mixed Müllerian tumor is a rare malignancy of the genital tract and extremely uncommon in extragenital sites. This report describes a case of malignant mixed Müllerian tumor arising in the lower peritoneum of a 72-year-old female patient. The patient presented with ascites, lower abdominal mass and pleural effusion. The serum level of CA125 was elevated. At operation a diffuse carcinosis associated with tumor mass measuring 20 × 15 × 10 cm in the vesicouterine and Duglas' pouch were found. The uterus and the adnexa were unremarkable. Histopathology revealed a typical malignant mixed Müllerian tumor, heterologous type. The epithelial component was positive for cytokeratin 7 and vimentin whereas the mesenchymal component was positive for Vimentin, S100 and focally for CK7. The histogenesis of this tumor arising from the peritoneum is still speculative. Based on the previous reports and the immunohistochemical analysis of our case, we believe that this is a monoclonal tumor with carcinoma being the "precursor" element. Nevertheless, further molecular and genetic evidence is needed to support such a conclusion.

## Background

Malignant mixed Müllerian tumor (MMMT) is a rare entity that arises from structures that are embryologically related to the Müllerian system [1,2]. The usual location of MMMT is the female genital tract. Extragenital origin is extremely rare [3-5]. Histologically and by immunohistochemistry, the tumor exhibits both epithelial and mesenchymal components.

Since the first report in 1955 by Ober and Black [5], to our knowledge there have been only 30 well documented reports of extragenital malignant mixed Müllerian tumors [6-15]. This prompted us to report on a tumor with primary peritoneal location and to review the relevant existing literature.

### Case Report

A 72-year-old woman, with unremarkable gynaecological history presented with chest pain and dyspnoea, increasing in intensity over the last three weeks. A chest X-ray showed pleural effusion and the subsequent fine needle aspiration cytology revealed malignant epithelial cells. The serum level of CA125 was 712 U/ml.

CT scan of abdomen and chest revealed ascites and pleural effusion but no tumor mass. Pelvic ultrasonography, however, revealed excrescences adjacent to the interior surface of the abdominal wall and tumor load in the lower part of abdomen. The uterus and the right ovary were described as normal; the left ovary was not visualised.

An ultrasound-guided biopsy from the tumor reported a carcinosarcoma (see later).

The patient underwent exploratory laparotomy. A widely spread peritoneal carcinosis and a tumor measuring 20 × 15 × 10 cm in the vesicouterine and Duglas' pouch were found.

Biopsy samples were taken from the tumor as well as from the serosa of the urinary bladder. Also, complete hysterectomy with partial omentectomy was performed. There was no suspicion of intrahepatic metastasis. Gallbladder, stomach, pancreas and appendix were unremarkable.

Histopathology was consistent with the diagnosis of a primary peritoneal malignant mixed Müllerian tumor given that the uterus and the adnexa were unremarkable.

Postoperatively, the patient underwent chemotherapy (Carboplatin in doses of 468 mg/360 mg every third week, as it was not felt that the patient was fit for more aggressive treatment). The disease progressed despite treatment and subsequent introduction of Treosulfan.

The patient passed away 12 months after diagnosis. No autopsy was performed.

## Material and methods

### Gross pathology

The tumor tissue submitted for pathology were fragmented, irregular tissue masses measuring altogether 16 × 12 × 4.5cm. All the fragments were tan-white, irregular fleshy masses with areas of necrosis and hemorrhage.

The uterus and the Fallopian tubes were unremarkable. The right and the left ovary were of normal dimensions. Numerous sections were taken from the tumor as well as from the uterus and adnexa.

### Histology and Histochemistry

The tissue was fixed in 10% neutral buffered formalin (pH 7,0), routinely processed, and embedded in paraffin using standard methods. Four-micrometer sections were stained with hematoxylin and eosin, Periodic acid-Schiff (PAS) with diastase predigestion, and Alcian/PAS.

### Immunohistochemistry

Formalin-fixed, paraffin-embedded tissue was stained with the peroxidase method using the EnVision visualisation system (Additional file 1).

## Results

### Histology and histochemistry

**Figure 1:**
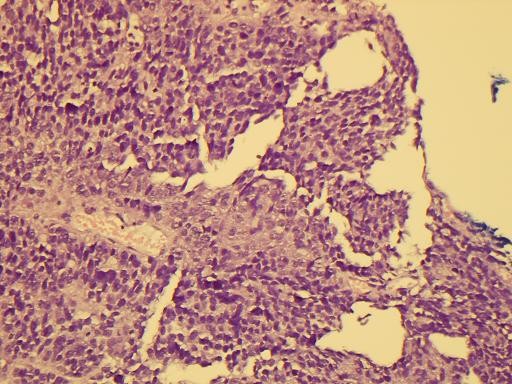
**Histological picture showing the admixed carcinoma and spindle cell sarcomatous elements (HE × 200)**.

**Figure 2:**
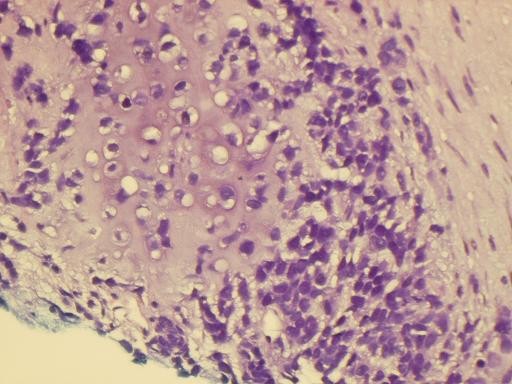
**Histological picture showing malignant cartilage admixed with sheets of undifferentiated spindle cells (HE × 400)**.

Most of the tumor tissue had the characteristics of poorly differentiated carcinoma (Figure 1). There was no squamous cell or glandular differentiation. The mesenchymal component was composed of sheets of undifferentiated spindle cells and areas of cartilage (Figure 2).

Numerous sections from the uterus and the adnexae showed no evidence of tumor.

### Immunohistochemistry

**Figure 3:**
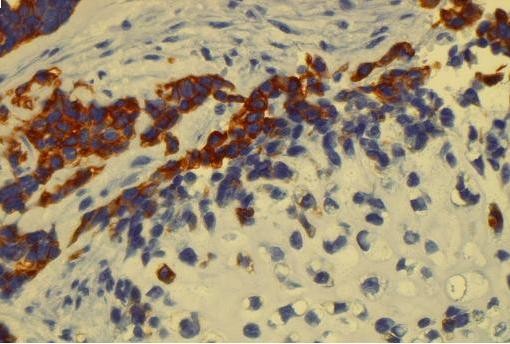
**Immunohistochemical stain with Cytokeratin 7 shows strong immunoreactivity of the undifferentiated epithelial cells (× 400)**.

**Figure 4:**
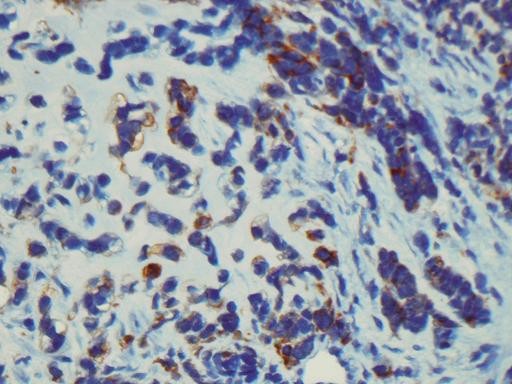
**Immunohistochemical stain with Cytokeratin 7 shows scattered Cytokeratin 7-positive cells in the mesenchymal component (× 400)**.

**Figure 5:**
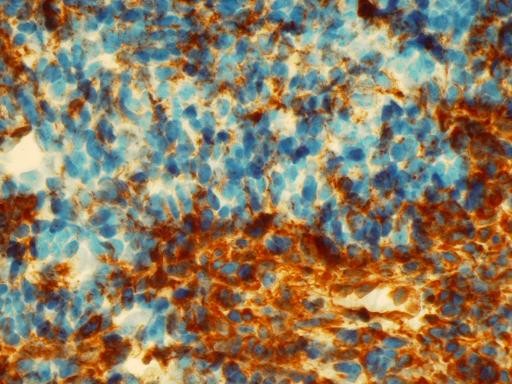
**Immunohistochemical stain with Vimentin shows areas with strong immunoreactivity of the epithelial and mesenchymal cells (× 400)**.

Immunohistochemical staining for cytokeratin 7 decorated cells of the epithelial component and scattered cells within the mesenchymal component (Figure 3 and Figure 4). Vimentin was strongly positive in mesenchymal component and sporadicaly in the epithelial areas (Figure 5). Cytokeratin 20 and calretinin were negative in both epithelial and mesenchymal elements.

## Discussion and the review of Literature

Malignant mixed Müllerian tumor (MMMT) is a highly aggressive tumor consisted of malignant epithelial and stromal cells. MMMTs were traditionally regarded as a subtype of uterine sarcomas or a mixture of true carcinoma and sarcoma, however several reports suggested a monoclonal origin of these tumors [1-3] Interestingly, molecular data published by Wada and co-workers [4] suggested that although most carcinosarcomas are combination tumors, some develop as collision tumors.

The morphology of the present tumor is consistent with malignant mixed Müllerian tumor. The epithelial component exhibited positivity for cytokeratin 7 and Vimentin and was negative for Cytokeratin 20. Moreover, the mesenchymal component was diffusely positive for Vimentin, focally for CK 7 and exhibited areas of heterologous malignant cartilage.

The primary peritoneal location and origin was confirmed after thorough gross and microscopic examination of the uterus and adnexae. Furthermore, absence of calretinin expression suggested that the tumor was of Müllerian rather than pure mesothelial origin [6]. A search of the literature revealed 30 previously reported cases of extragenital MMMT (Additional file 2) with the majority of the patients being in the postmenopausal age [5-19]. Twenty-two of the reported cases were of primary peritoneal origin and most of them arose in the pelvis [19]. Thirteen were heterologous type [19].

In the homologous type MMMT, mesothelioma would rightfully be considered as a possibility [6]. However, in this case a positive reaction of the cells to Calretinin would be expected [6]. Immunoreactivity of the epithelial cells to Cytokeratin 7 and negative reaction for cytokeratin 20 also points toward Müllerian origin. A theoretical possibility is of course the origin in endometriosis or endosalpingiosis. However, although impossible to rule out, the lack of demonstrable endometriosis associated with the patient's current tumor makes this hypothesis unlikely [16].

Several theories have emerged in the attempt to explain the biphasic appearance of the tumor, the most important of which are the "collision", "conversion" and the "combination" theory [1]. Sternberg et al. [20] was the first to suggest the conversion, stating that sarcomatous elements may develop from carcinoma. They described a case of metastatic heterologous type carcinosarcoma of the omentum with primary endometrial origin. There was no evidence of sarcoma component in the primary tumor. From that time onward, a number of cases with metachronous or synchronous gynaecologic carcinoma have been reported [11,14,16]. Masuda et al. [21] further supported the conversion theory with their study in which cell lines established from malignant mixed Müllerian tumors showed the ability of the epithelial tumor cells to transform into epithelial, mesenchymal or both types of differentiation in vitro, while the mesenchymal cells did not show similar capabilities.

MMMT has a poor prognosis with most of the patients following a rapidly fatal course regardless of the initial tumor stage [9]. A review done by Garamvoelgyi et al. [14] showed that most patients passed away within one year with median postoperative survival time being 14 months. Due to the rarity of the disease, limited data regarding the management of peritoneal MMMT exist. Treatment modalities include surgery, chemotherapy and irradiation with various survival outcomes. Ko et al. [19] reported on a patient that was treated with optimal tumor debulking and a combination of chemotherapy with Ifosfamide and Cisplatin, followed by pelvic irradiation. There were no signs of recurrence for 48 months and was the case with the longest disease-free survival in the reported literature.

## Conclusion

Based on the ample evidence of the previous reports, and the immunohistochemical analysis of our case, we believe that this is a monoclonal tumor with the carcinoma being the "precursor" element. Nevertheless, further molecular and genetic evidence is needed to support such a conclusion.

## Authors' contributions

FK prepared the manuscript and reviewed the literature. HRH provided the clinical data. VRH reviewed the slides, LGL reviewed the manuscript, TH reviewed the slides and supervised the preparation of the manuscript.

## Competing interests

The authors declare that they have no competing interests.

## Consent

Informed consent was obtained from the patient for publication of this case report and accompanying images.

## Supplementary Material

Additional file 1 **Immunohistochemical stains**. Click here for file

Additional file 2 **Primary peritoneal MMMT reported in the literature**. Click here for file

## References

[B1] McCluggage WG Malignant biphasic uterine tumours: carcinosarcomas or metaplastic carcinomas? J Clin Pathol 2002 55 321 325 11986333 10.1136/jcp.55.5.321 PMC1769650

[B2] Mayall F Rutty K Campbell F Goddard H p53 immunostaining suggests that uterine carcinosarcomas are monoclonal Histopathology 1994 24 3 211 214 10.1111/j.1365-2559.1994.tb00512.x 8200622

[B3] Watanabe M Shimizu K Kato H Imai H Nakano H Sugawa M Shiraishi T Carcinosarcoma of the uterus: immunohistochemical and genetic analysis of clonality of one case Gynecol Oncol 2001 82 3 563 567 10.1006/gyno.2001.6307 11520156

[B4] Wada H Enomoto T Fujita M Yoshino K Nakashima R Kurachi H Haba T Wakasa K Shroyer KR Tsujimoto M Hongyo T Nomura T Murata Y Molecular evidence that most but not all carcinosarcomas of the uterus are combination tumors Cancer Res 1997 57 5379 5385 9393763

[B5] Ober WB Black MB Neoplasms of the subcoelomic mesenchyme: report of two cases Arch Pathol 1955 59 698 705 14375493

[B6] Sumathi VP Murnaghan M Dobbs SP McCluggage WG Extragenital Müllerian carcinosarcoma arising from the peritoneum: report of two cases Int J Gynecol Cancer 2002 12 764 767 10.1046/j.1525-1438.2002.01146.x 12445257

[B7] Lauchlan SC The secondary Müllerian system Obstet Gynecol Surg 1972 27 133 146 10.1097/00006254-197203000-00001 4614139

[B8] Zelmanowicz A Hildesheim A Sherman ME Sturgeon SR Kurman RJ Barrett RJ Berman ML Mortel R Twiggs LB Wilbanks GD Brinton LA Evidence for a common etiology for endometrial carcinomas and malignant mixed Müllerian tumors Gynecol Oncol 1998 69 253 257 10.1006/gyno.1998.4941 9648597

[B9] Friedrich M Villena-Heinsen C Mink D Bonkhoff H Schmidt W Carcinosarcoma, endometrial intraepithelial carcinoma and endometriosis after tamoxifen therapy in breast cancer Eur J Obstet Gynecol 1999 82 85 87 10.1016/S0301-2115(98)00166-3 10192492

[B10] Nascimento MC Choo PS Bligh J Obermair A Primary peritoneal malignant mixed Müllerian tumor (MMMT): a case report Cancer Therapy 2004 2 571 574

[B11] Shen DH Khoo US Xue WC Ngan HYS Wang JL Liu VWS Chan YK Cheung ANY Primary peritoneal malignant mixed Müllerian tumors: a clinicopathologic, immunohistochemical and genetic study Cancer 2001 91 1052 1060 10.1002/1097-0142(20010301)91:5<1052::AID-CNCR1097>3.0.CO;2-A 11251959

[B12] Shintaku M Matsumoto T Primary Müllerian carcinosarcoma of the retroperitoneum: report of a case International J Gynecol Pathol 2001 20 191 195 10.1097/00004347-200104000-00013 11293167

[B13] Ibanez-Manlapaz IG McCoy D Vincent V III Ule UJ Malignant mixed Müllerian tumor of the extra-genital coelomic epithelium: report of two cases Pathology Oncology Research 1997 3 130 134 10.1007/BF02907808 11173640

[B14] Garamvoelgyi E Guillou I Gebhard S Salmeron M Seematter RJ Hadji MH Primary malignant mixed Müllerian tumor (metaplastic carcinoma) of the female peritoneum Cancer 1994 74 854 863 10.1002/1097-0142(19940801)74:3<854::AID-CNCR2820740311>3.0.CO;2-R 7518735

[B15] Choong SYM Scurry JP Planner RS Grant PT Extrauterine malignant mixed Müllerian tumor of primary peritoneal origin Pathology 1994 26 497 498 10.1080/00313029400169272 7892058

[B16] Garde JR Jones MA McAfee R Tarraza HM Extragenital malignant mixed Müllerian tumor Gynaecol Oncol 1991 43 186 190 10.1016/0090-8258(91)90070-L 1660435

[B17] Weisz-Carrington P Bigelow B Schinella RA Extragenital mixed heterologous tumor of Müllerian type arising in the cecal peritoneum: report of a case Dis Colon Rectum 1977 20 4 329 333 10.1007/BF02586432 193674

[B18] Hasiuk AS Petersen RO Hanjani P Griffin TD Extragenital malignant mixed Müllerian tumor: case report and review of the literature Am J Clin Pathol 1984 81 102 105 6318547 10.1093/ajcp/81.1.102

[B19] Ko ML Jeng CJ Huang SH Shen J Tzeng CR Chen SC Primary peritoneal carcinosarcoma (malignant mixed Müllerian tumor): Report of a case with five-year disease free survival after surgery and chemoradiation and a review of literature Acta Oncologica 2005 44 7 756 760 10.1080/02841860500252016 16227168

[B20] Sternberg WH Clark WH Smith RC Malignant mixed Müllerian tumor (malignant mixed mesodermal tumor) of the uterus: a study of 21 cases Cancer 1954 7 704 724 10.1002/1097-0142(195407)7:4<704::AID-CNCR2820070411>3.0.CO;2-D 13172686

[B21] Masuda A Takeda A Fukami H Yamada C Matsuyama M Characteristics of cell lines established from mixed mesodermal tumor of the human ovary: carcinomatous cells are changeable to sarcomatous cells Cancer 1987 60 2969 2703 10.1002/1097-0142(19871201)60:11<2696::AID-CNCR2820601120>3.0.CO;2-R 2445463

