# Supplementary material for: Primary malignant mixed müllerian tumor of the peritoneum a case report with review of the literature
Source: World J Surg Oncol. 2011 Feb 4;9:17. doi: 10.1186/1477-7819-9-17 (PMC3039619; doi:10.1186/1477-7819-9-17)
Supplement: Additional file 1 — Immunohistochemical stains. [file 1477-7819-9-17-S1.DOC]

| **Table 1.** Immunohistochemical stains | | |  |  |
| --- | --- | --- | --- | --- |
| **Antibody** | **Clone** | **Source** | **Dilution** | **Pretreatment** |
| Cytokeratin 7 | OV-TL12/30 | Dako | 1;2000 | MTE* |
| Cytokeratin 20 | KS20.8 | Dako | 1;100 | MTE |
| Vimentin | Vim3B4 | Dako | 1;400 | MTE |
| S100 | Polyclonal | Dako | 1;5000 | Protease^ |
| CEA | Polyclonal | Dako | 1;5000 | MTE |
| Calretinin | DAKCalret1 | Dako | 1;100 | MTE |
| *Microwave treatment in Tris EDTA, pH 9.0 | | | |  |
| ^Protease digestion | |  |  |  |
