# Supplementary material for: Primary malignant mixed müllerian tumor of the peritoneum a case report with review of the literature
Source: World J Surg Oncol. 2011 Feb 4;9:17. doi: 10.1186/1477-7819-9-17 (PMC3039619; doi:10.1186/1477-7819-9-17)
Supplement: Additional file 2 — Primary peritoneal MMMT reported in the literature. [file 1477-7819-9-17-S2.DOC]

**Table 2**. Primary peritoneal MMMT reported in the literature

| **Case** | **Year of Report** | **Authors** | **Age** | **Site** | **Diagnosis** |
| --- | --- | --- | --- | --- | --- |
| 1 | 1955 | Ober and Black | 74 | Pelvic peritoneum | MMMT, homologous |
| 2 | 1967 | Ferrie and Ross | 47 | Abdominal retroperitoneum | MMMT, homologous |
| 3 | 1977 | Weiss-Carrington et al. | 77 | Cecal peritoneum | MMMT, heterologous |
| 4 | 1982 | Marchevsky et al. | 40 | Cul-de-sac peritoneum | MMMT, homologous |
| 5 | 1983 | Herman and Tessler | 72 | Abdominal posterior peritoneum | MMMT, heterologous |
| 6 | 1984 | Hasiuk et al. | 77 | Abdominal posterior peritoneum | MMMT, heterologous |
| 7 | 1986 | Chumas et al. | 67 | Rectal peritoneum | MMMT, homologous |
| 8 | 1986 | Campins et al. | 58 | Pelvic peritoneum | MMMT, homologous |
| 9 | 1987 | Chen and Wolk | 52 | Pelvic peritoneum | MMMT, homologous |
| 10 | 1989 | El-Jabbour | 76 | Ascending colon peritoneum | MMMT, heterologous |
| 11 | 1989 | Ohno et al. | 66 | Descending sigmoid colon peritoneum | MMMT, heterologous |
| 12 | 1990 | Fenoglio-Preiser et al. | ? | Cecal peritoneum | MMMT, heterologous |
| 13 | 1991 | Solis et al. | 54 | Cul-de-sac peritoneum | MMMT, heterologous |
| 14 | 1991 | Garde et al. | 65 | Diaphragm peritoneum | MMMT, heterologous |
| 15 | 1993 | Nimaroff et al. | 82 | Sigmoid colon peritoneum | MMMT, homologous |
| 16 | 1994 | Garamvoelgyi et al. | 59 | Pelvic peritoneum | MMMT, heterologous |
| 17 | 1994 | Garamvoelgyi et al. | 64 | Pelvic peritoneum cul-de-sac | MMMT, homologous |
| 18 | 1994 | Garamvoelgyi et al. | 84 | Uterine subserosa | MMMT, heterologous |
| 19 | 1994 | Choong et al. | 63 | Serosa of the sigmoid colon | MMMT |
| 20 | 1995 | Mira et al. | 62 | Pelvic peritoneum | MMMT, heterologous |
| 21 | 1995 | Mira et al. | 83 | Cecal peritoneum | MMMT, heterologous |
| 22 | 1997 | Rose et al. | 57 | Cul-de-sac peritoneum | MMMT, homologous |
| 23 | 1997 | Rose et al. | 71 | Peritoneum of liver and other surfaces | MMMT, homologous |
| 24 | 1997 | Rose et al. | 67 | Omentum and peritoneum | MMMT, homologous |
| 25 | 2001 | Shintaku and Matsumoto | 51 | Retroperitoneum lateral pelvic wall | MMMT, heterologous |
| 26 | 2002 | Sumathi et al. | 77 | Pelvic peritoneum | MMMT, heterologous |
| 27 | 2002 | Sumathi et al. | 87 | Omentum, pelvic peritoneum | MMMT, heterologous |
| 28 | 2002 | Dincer et al. | 50 | Pelvic peritoneum | MMMT, heterologous |
| 29 | 2002 | Wei et al. | 67 | Omentum | MMMT, heterologous |
| 30 | 2005 | Ma-Lee Ko et al. | 45 | Pelvic peritoneum, cul-de-sac | MMMT, homologous |
| 31 | 2010 | Current study |  | Pelvic peritoneum | MMMT, heterologous |
